# Channel Effects on Online Health Information Seeking in the Age of AI: An Extension of the CMIS Framework

**DOI:** 10.3390/bs16071137

**Published:** 2026-07-07

**Authors:** Heyang Zhang, Kexin Tai, Yueqin Hu

**Affiliations:** Faculty of Psychology, Beijing Normal University, Beijing 100875, China; heyang.zhang@mail.bnu.edu.cn (H.Z.);

**Keywords:** online health information-seeking behavior, information channels, source credibility, artificial intelligence (AI), short-video

## Abstract

The rapid expansion of online health information channels, particularly emerging artificial intelligence (AI) platforms, is transforming how individuals access and evaluate health information. Drawing on an extended Comprehensive Model of Information Seeking (CMIS), this research examined how different channel types (AI-based, short-video, and text-based) influence online health information-seeking behavior (OHISB) through a pilot validation (*N* = 258), a cross-sectional survey (Study 1; *N* = 300), and a between-subjects experiment (Study 2; *N* = 300). Study 1 tested an extended CMIS model incorporating channel type, source credibility, information credibility, and perceived usefulness, while Study 2 examined the causal effects of channel exposure. Structural equation modeling in Studies 1 and 2 consistently showed that source and information credibility predicted OHISB indirectly through perceived usefulness. AI channels showed no advantage in Study 1, whereas Study 2 found that participants perceived AI sources as more credible and useful, which indirectly predicted stronger intentions for SAMC and information seeking through the credibility–usefulness pathway. This change may reflect methodological differences between self-report recall-based and direct exposure designs, and the public’s growing familiarity with AI technologies. By integrating channel characteristics and credibility perceptions, this study extends the CMIS framework and provides evidence for AI’s enhanced perceived credibility in health information contexts, offering insights for improving AI-driven health communication.

## 1. Introduction

The rapid development of internet technology has established various online channels as primary sources for health information acquisition. Concurrently, rising public health awareness has made online health information-seeking behavior (OHISB) increasingly prevalent, as individuals turn to diverse digital platforms to access health information for self-management and informed decision-making. OHISB encompasses a range of behaviors through which people seek social support, look for alternatives or supplements to medical care, and obtain information to better understand their health (Popovac & Roomaney, 2022). However, information available through different online channels varies considerably in quality, credibility, and usefulness, posing challenges for individuals seeking reliable health knowledge.

Past research has examined the relationship between information channels and OHISB (Cao et al., 2016). Nonetheless, the digital landscape has evolved dramatically since these investigations. The emergence of short-video platforms and the recent, rapid advancement of generative AI chatbots represent new and influential channel types. These channels differ markedly from traditional text-based sources (e.g., search engines, forums) in their interface, information presentation modality (e.g., text, video, interactive dialog), and perceived authority. For instance, AI platforms can synthesize information from multiple sources interactively, whereas short-video platforms offer visually engaging explanations. Such channel differences may shape users’ cognitive evaluations, such as perceived credibility and usefulness, ultimately influencing their engagement in OHISB. Therefore, a fresh investigation into how these modern channels affect OHISB is critically needed.

Recent empirical research has begun to document this shift toward AI-driven health information channels. A comprehensive scoping review by Bautista et al. (2025) examined health consumers’ use and perceptions of generative AI chatbots, revealing growing adoption but also highlighting concerns about credibility assessment. Svestkova et al. (2025) identified trust as a critical predictor of willingness to use generative AI for health information, while Guo et al. (2025) demonstrated that perceived usefulness and trust significantly influence intentions to adopt ChatGPT-generated health information. Lund et al. (2025) provided an integrative review synthesizing AI and information-seeking research, and Yun and Bickmore (2025) documented the evolving landscape where LLM-based chatbots are increasingly competing with traditional search engines and health websites. These studies collectively support the notion that AI platforms are becoming primary channels for health information seeking, while also highlighting the importance of credibility perceptions in differentiating these sources—a central focus of the present research.

### 1.1. A Combined Framework of Information Seeking and Adoption

The Comprehensive Model of Information Seeking (CMIS) provides a foundational framework for understanding how antecedents and information carrier factors influence information-seeking behaviors (Johnson & Meischke, 1993). The model posits that demographic characteristics and direct experience, as antecedents, affect individuals’ perceptions of information carriers in terms of their utility and credibility, which ultimately drive information-seeking behavior. Given its focus on both information carriers and behavioral outcomes, the CMIS is highly applicable to the context of online health information seeking.

To further elucidate the cognitive mechanisms that occur after individuals encounter information, this study draws on the Information Adoption Model (IAM) (Sussman & Siegal, 2003). Derived from the Elaboration Likelihood Model, the IAM proposes that information quality (central route) and source credibility (peripheral route) influence adoption intentions through the mediating role of perceived usefulness. This model highlights the evaluative processes through which individuals assess and decide whether to adopt information, providing a cognitive complement to the behavioral orientation of the CMIS.

Integrating these two perspectives, we propose to combine the CMIS’s focus on behavioral antecedents with the IAM’s emphasis on cognitive mediation. In the extended framework, credibility and utility are conceptualized as a sequential rather than parallel process, reflecting how individuals’ evaluations of credibility inform their judgments of utility, which subsequently drive seeking behavior. The model distinguishes between source credibility (the perceived trustworthiness of the information platform or channel) and information credibility (the perceived trustworthiness of the specific content) (Metzger et al., 2003). This distinction enables a more nuanced examination of how different aspects of credibility shape perceptions of usefulness and motivate individuals to seek or act upon health information in online environments.

### 1.2. Extending the Framework: Information Channels and Individual Antecedents

Building on the combined CMIS–IAM framework, this study further incorporates contextual and individual factors that influence online health information seeking, with a primary focus on channel characteristics. Specifically, we examine three major types of information channels: AI-based, short-video, and text-based platforms. These channels differ fundamentally in their modality, that is, the way information is presented (e.g., text, interactive dialog, or video). According to Dual-Coding Theory (Paivio, 1971/2013), multimodal information (e.g., video combining audio and text) engages both visual and verbal processing systems, which may enhance comprehension, recall and persuasion. This perspective provides a theoretical basis for expecting that different channels may elicit distinct evaluations of credibility and usefulness.

While channel features are central to this study, two individual factors (eHealth literacy and health anxiety) are considered control variables together with demographic factors to account for potential individual differences. eHealth literacy refers to the ability to seek, evaluate, and apply health information from electronic sources (Norman & Skinner, 2006), whereas health anxiety reflects excessive preoccupation with illness due to misinterpretation of bodily sensations (Salkovskis et al., 2002). These variables may influence how individuals process online information and evaluate its credibility, but they are not the focal predictors in the present framework.

### 1.3. The Present Study

Building on the extended CMIS–IAM framework, the present research aims to empirically examine how different types of online information channels influence online health information-seeking behavior (OHISB). Despite their utility, the CMIS and IAM were developed prior to the rise of contemporary digital platforms, particularly AI-based and short-video applications. Consequently, an integrated model that incorporates channel type as a key antecedent, differentiates its effects on source credibility (the perceived trustworthiness of the platform) and information credibility (the perceived trustworthiness of the content), and examines their distinct pathways to OHISB via perceived usefulness is notably absent from the literature.

Furthermore, while the multidimensional OHISB framework proposed by Popovac and Roomaney (2022) provides a robust conceptual basis for understanding online health engagement, its factor structure and validity in Chinese samples require further empirical verification. To address this gap and to examine how information channels influence OHISB, we adopted a multi-study, multi-method approach. First, the pilot validation (Appendix A) translated and validated a Chinese version of the OHISB scale. Next, Study 1 employed a cross-sectional survey to test the proposed extended CMIS model, examining the relationships among channel type, dual credibility perceptions, perceived usefulness, and OHISB. To establish causal evidence for channel effects, Study 2 adopted an experimental design that manipulated channel exposure (AI-based vs. short-video vs. text-based) while holding information content constant.

**Figure 1:**
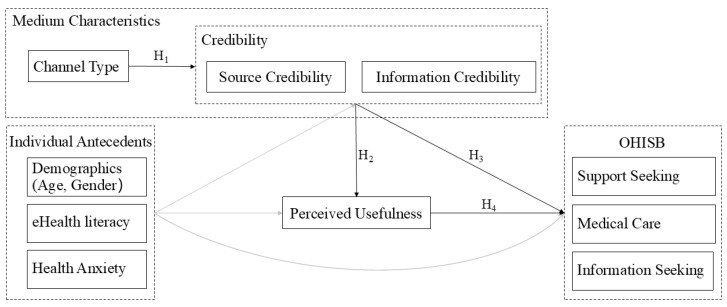
Conceptual Model of the Extended Comprehensive Model of Information Seeking. Note. Black arrows represent the hypothesized relationships among channel type, credibility evaluations, perceived usefulness, and online health information-seeking behavior (OHISB). Gray arrows represent covariate controls of age, gender, eHealth literacy and health anxiety on perception and behavioral measures.

The overall conceptual model and the four hypotheses are presented in Figure 1.

**H_1_.**  *Channel type significantly influences perceptions of source credibility and information credibility.*

**H_2_.**  *Source credibility and information credibility positively influence perceived usefulness.*

**H_3_.**  *Source credibility and information credibility directly influence OHISB.*

**H_4_.**  *Perceived usefulness positively influences OHISB.*

Additionally, eHealth literacy and health anxiety were included as control antecedents to account for individual differences in evaluating online health information. By testing these hypotheses, this study aims to extend the CMIS framework by incorporating channel characteristics and credibility perceptions, thereby offering insights into optimizing digital health communication across emerging platforms.

## 2. Study 1: Testing the Extended CMIS Model

### 2.1. Method

#### 2.1.1. Participants and Procedure

Study 1 used a new sample drawn from the same general population as the scale validation described in Appendix A to validate the measurement model and test the hypothesized structural relationships in the conceptual model. Participants were all working adults or retirees rather than students, as these groups are more likely to experience health concerns and engage in online health information seeking. The questionnaire was distributed through the Credamo Online Data Platform, yielding 408 responses. Participants were randomly assigned to receive one version of the questionnaire corresponding to one of the three channel conditions (text-based, short-video, or AI-based). After excluding failures on attention checks, speeders, and ineligible cases, 300 valid responses were retained. Participants were from 28 provinces or municipalities across China, ensuring adequate representativeness. The sample included 196 women (65.3%) and 104 men (34.7%), aged 20 to 65 years (*M* = 30.13 years, *SD* = 7.60), and 89% held a bachelor’s degree or higher.

#### 2.1.2. Measures

**Online Health Information-Seeking Behavior (OHISB).** OHISB was measured with a 20-item scale comprising three dimensions: Support Seeking (SS), Supplement or Alternative to Medical Care (SAMC), and information seeking (IS). The scale was adapted for the Chinese context using translation and back-translation procedures. Details regarding its psychometric properties, including the full item list and validation summary, are provided in Appendix A. To examine the role of information channels, three parallel versions were designed for text-based, short-video, and AI-based channels. Items were identical across versions except that channel references were substituted (e.g., “online forums,” “short-video platforms,” or “AI assistants”).

**Electronic Health Literacy.** Electronic health literacy was measured using the eHealth Literacy Scale (eHEALS; Neter & Brainin, 2012), which is widely used in online health information research. The scale consists of 4 items (e.g., I know how to use online channels to find health information that is relevant to me.) rated on a 7-point Likert scale (1 = strongly disagree, 7 = strongly agree). Higher scores indicate greater perceived ability to search for, evaluate, and apply online health information.

**Health Anxiety.** Health anxiety was measured using the Chinese Short Health Anxiety Inventory (CSHAI; Salkovskis et al., 2002; Zhang et al., 2015). The CSHAI contains 18 items (e.g., I find it difficult to control my thoughts about illness.) rated on a 4-point scale (1 = never, 4 = always). Higher scores reflect greater anxiety related to health concerns.

**Source Credibility and Information Credibility.** Source credibility was assessed using four items adapted from Cheng et al. (2021) and Kumar et al. (2023), assessing perceptions of a source’s trustworthiness and expertise (e.g., The information source is reliable.). Information credibility was measured using three items from the Information Reliability Scale developed by Flanagin and Metzger (2000), evaluating perceived accuracy and reliability of health information (e.g., How accurate do you think the information is.). Both scales used 7-point Likert ratings (1 = strongly disagree, 7 = strongly agree), with higher scores reflecting greater perceived credibility.

**Perceived Usefulness.** Perceived usefulness was measured using items adapted from Cheung et al. (2008), assessing the extent to which individuals view online health information as personally relevant and beneficial (e.g., The health information provided by this online channel offers me professional health knowledge.). Items were rated on a 7-point Likert scale (1 = strongly disagree, 7 = strongly agree). Higher scores represent greater perceived usefulness.

#### 2.1.3. Data Analysis

All analyses were conducted using R version 4.4.2. Confirmatory factor analysis was first performed to validate the 20-item OHISB structure using a different sample. Then, descriptive statistics, reliability coefficients, and bivariate correlations were computed for all study variables to assess internal consistency and preliminary associations. Differences across the three channel conditions (text-based, short-video, AI-based) in perceived credibility, usefulness, and OHISB were examined using one-way ANOVA with Tukey’s HSD post hoc comparisons. Structural equation modeling was used to test the hypothesized relationships among source credibility, information credibility, perceived usefulness, and OHISB. Model fit was evaluated using the same indices reported in Appendix A (*χ*^2^/*df*, CFI, TLI, RMSEA, SRMR).

### 2.2. Results

#### 2.2.1. Confirmatory Factor Analysis

The CFA model on 20 items demonstrated acceptable fit, *χ*^2^ (167) = 321.14, *χ*^2^/*df* = 1.92, CFI = 0.939, TLI = 0.931, RMSEA = 0.055 [0.046, 0.065], and SRMR = 0.064. All factor loadings were significant (*p* < 0.001), providing strong support for the construct validity of the adapted scale. These results confirm that the factor structure was stable and replicable across independent samples.

#### 2.2.2. Descriptive Statistics and Correlations

The means, standard deviations, internal consistency coefficients, and correlations among the study variables are presented in Table 1. As shown, source credibility, information credibility, and perceived usefulness were all positively associated with OHISB and its subscales (SS, SAMC, and IS).

#### 2.2.3. Channel Effects on Credibility, Perceived Usefulness and OHISB

**Figure 2:**
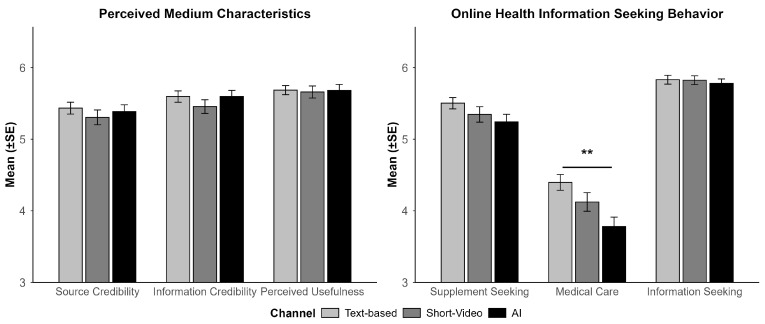
Mean and SE in Credibility, Usefulness and OHISB Across Channels (Study 1). Note. Bars represent mean scores (±1 SE). Significance markers indicate omnibus ANOVA main effects. ** *p* < 0.01.

A series of one-way ANOVAs examined the effects of information channel (text-based, short-video, AI) on all study variables and are presented in Table 2 and Figure 2. No significant channel differences emerged for source credibility, information credibility, perceived usefulness, SS, or IS (all *ps* > 0.16). However, there was a significant main effect of channel on SAMC, *F* (2, 297) = 6.18, *p* = 0.002, *η*^2^ = 0.040. Tukey HSD post hoc comparisons indicated that participants in the text-based condition reported higher SAMC intentions than those in the AI condition (MD = 0.615, *p* = 0.002, 95% CI [0.202, 1.028]). This pattern suggests that participants exposed to traditional text-based health information were more likely to seek alternative medical advice than those exposed to AI-based materials.

#### 2.2.4. Structural Equation Modeling

**Figure 3:**
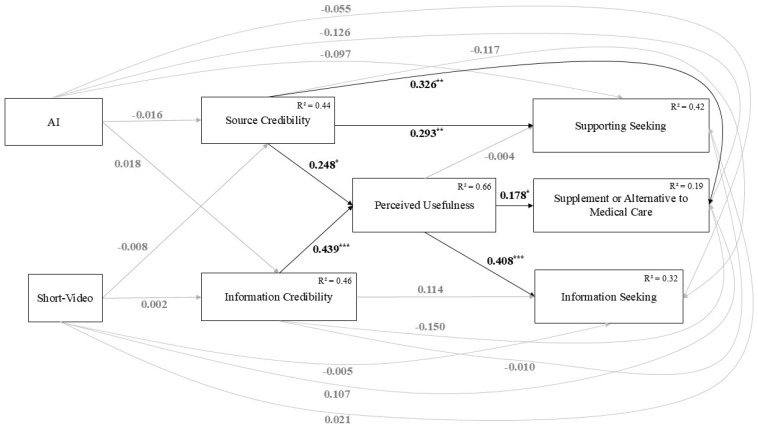
Structural Equation Model of Channel, Credibility, Usefulness and OHISB (Study 1). Note. Values represent standardized path coefficients. Black lines indicate significant paths (*p* < 0.05), while gray lines indicate non-significant paths (*p* ≥ 0.05). Covariances among variables were estimated but not depicted in the figure to maintain clarity. Text-based was the reference channel. Age, gender, eHealth literacy, and health anxiety were controlled in the model. * *p* < 0.05. ** *p* < 0.01. *** *p* < 0.001.

A structural equation model (Figure 3) was estimated to examine the hypothesized paths from credibility to usefulness and behavioral outcomes, controlling for gender, age, eHealth literacy, and health anxiety. The model fit data well, *χ*^2^ (2) = 2.13, *χ*^2^/*df* = 1.07, CFI = 1.000, TLI = 0.997, RMSEA = 0.015 [0.000, 0.116], and SRMR = 0.004.

Table 3 shows the standardized path coefficients. Both source credibility and information credibility significantly predicted perceived usefulness (*β* = 0.248, *p* = 0.013; *β* = 0.439, *p* < 0.001, respectively), which in turn predicted SAMC (*β* = 0.178, *p* = 0.032) and IS (*β* = 0.408, *p* < 0.001). The direct effect of source credibility on SS (*β* = 0.293, *p* = 0.004) and the direct effect of source credibility on SAMC (*β* = 0.326, *p* = 0.001) were also significant. No significant direct channel effects on information credibility or behavioral outcomes were observed. These findings supported perceived usefulness as the central mediator linking credibility perceptions to OHISB.

## 3. Study 2: An Experimental Study on Channel Manipulation

While Study 1 supported the extended CMIS model, it did not detect significant channel effects. This may reflect a limitation: participants were asked to recall or imagine seeking health information through a given channel, engaging memory-based reconstruction rather than capturing immediate reactions to standardized content. Methodological research indicates that recall-based assessments and direct exposure paradigms can yield divergent results (Schwarz, 2007). Moreover, the recalled information might vary substantially across participants, making the content not directly comparable across conditions.

To overcome these limitations, Study 2 adopted an experimental design that directly manipulated channel type while holding information content constant. Each participant was exposed to identical health information presented through one of the three channels (text-based, short-video, or AI). This approach enabled a more rigorous test of whether the same information on different communication channels would elicit different levels of perceived source credibility, information credibility, perceived usefulness, and subsequent information-seeking intentions. Data were collected during a period of heightened public awareness of AI systems such as DeepSeek, when participants were likely more familiar with AI and viewed it as a more authoritative and competent source. This temporal distinction—Study 1 conducted prior to widespread AI familiarity versus Study 2 following AI’s proliferation into public awareness—offers an opportunity to examine channel effects under different technological exposure conditions.

### 3.1. Method

#### 3.1.1. Participants and Procedure

Participants were recruited from the same general population as in Study 1 but represented an independent sample. Eligible participants were adults who were currently employed or retired, fluent in Chinese, and had prior experience using all of the following methods to access online health information: text-based platforms, short-video applications, or AI tools. Exclusion criteria included full-time students and individuals working in medical professions.

A priori power analysis using G*Power 3.1.9.7 indicated that a minimum of 159 participants was needed to detect a medium effect size (*f* = 0.25) with *α* = 0.05 and 1 − *β* = 0.80. Data were collected via the Credamo online platform. Of the 333 total responses received, 300 valid cases were retained after excluding those who failed attention checks, responded unrealistically quickly, or did not meet inclusion criteria (effective rate = 90.1%). The final sample consisted of 200 women (66.7%) and 100 men (33.3%), aged 20 to 65 years (*M* = 30.13 years, *SD* = 7.60), with 88% holding a bachelor’s degree or higher. Participants were drawn from 27 provinces and 120 cities across China. The majority were employed in the private sector (60%), covering 19 different industries, providing broad demographic and occupational diversity.

#### 3.1.2. Stimuli and Manipulation

Study 2 adopted a between-subject experimental design in which participants were randomly assigned to one of three channel conditions: text-based, short-video, or AI. The goal was to examine whether exposure to different types of health information channels influences perceived credibility, usefulness, and online health information-seeking behaviors.

The experimental materials consisted of simulated web interfaces depicting online health information on two common topics: “Can ibuprofen and aspirin be taken together?” and “How should acute gastroenteritis be treated?” Participants viewed and could scroll through these interfaces as they would on actual platforms. The textual content was identical across conditions to ensure that any observed effects could be attributed to channel differences rather than message content. In the text-based condition, participants viewed static but scrollable simulations resembling discussion posts or articles typically found on text-sharing platforms. In the short-video condition, participants viewed simulated short-video app interfaces displaying health-related video thumbnails and brief descriptions. In the AI condition, participants saw a chatbot-style conversational interface showing AI-generated responses to the same health questions, featuring dialog bubbles and interactive formatting. Supplementary Materials contain annotated screenshots documenting these interfaces for replication purposes. While the interfaces were interactive (scrollable, realistic layout), they did not include full back-end functionality (e.g., real-time AI generation), balancing ecological validity with experimental control over content equivalence.

After viewing their assigned stimuli, participants completed a manipulation-check item confirming which channel type they had seen (text, video, or AI). They then filled out the questionnaire assessing source credibility, information credibility, perceived usefulness, health anxiety, and online health information-seeking behaviors.

To ensure comparability across conditions, items from the online health information-seeking behavior (OHISB) scale were adapted to explicitly reference the respective channel (e.g., “When using short-video platforms…”, “When consulting AI tools…”). Because AI-based channels do not involve social support functions, the Support-Seeking subscale was omitted, retaining only the Supplement or Alternative to Medical Care (SAMC) and information seeking subscales.

#### 3.1.3. Measures

All variables were measured using the same validated instruments as in Study 1, including eHealth literacy, health anxiety, source credibility, information credibility, perceived usefulness, and the adapted online health information-seeking behavior scale. The internal consistency coefficients (Cronbach’s α) for all scales in this study can be found in Table 4, ranging from 0.70 to 0.93. Most scales demonstrated satisfactory to excellent reliability. The lowest value (eHealth literacy, α = 0.70) is acceptable for research purposes, and we retained this measure as a covariate.

#### 3.1.4. Data Analysis

All analyses were conducted using R version 4.4.2. Descriptive statistics and internal consistency coefficients were computed to assess data quality and reliability. Next, one-way analyses of variance were performed to examine the main effects of information channel on the dependent variables. Tukey’s HSD post hoc tests were used to probe pairwise differences among channel types. Finally, structural equation modeling was conducted to test the hypothesized mediation model linking information channel to behavioral outcomes through perceived credibility and usefulness. Model fit was assessed as in Study 1.

### 3.2. Results

#### 3.2.1. Descriptive Statistics and Reliability

Table 4 presents the descriptive statistics, internal consistencies, and correlations among all study variables, including two covariates, eHealth literacy and health anxiety. Both covariates were positively associated with credibility perceptions and perceived usefulness, supporting their inclusion as control variables.

#### 3.2.2. Channel Effects on Credibility, Perceived Usefulness and OHISB

**Figure 4:**
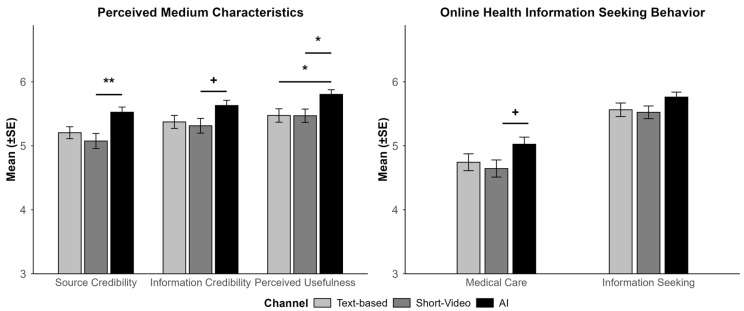
Mean and SE in Credibility, Usefulness and OHISB Across Channels (Study 2). Note. Bars represent mean scores (±1 SE). Significance markers indicate omnibus ANOVA main effects: + *p* < 0.10. * *p* < 0.05. ** *p* < 0.01.

A one-way analysis of variance was conducted with information channel (text-based, short-video, AI) as a between-subject factor. The ANOVA results and group means are presented in Table 5 and Figure 4. Using text-based as the reference, the results revealed significant channel effects on source credibility, *F* (2, 297) = 5.56, *p* = 0.004, *η*^2^ = 0.036, and perceived usefulness, *F* (2, 297) = 4.07, *p* = 0.018, *η*^2^ = 0.027. The channel effect on information credibility was of marginal significance, *F* (2, 297) = 2.81, *p* = 0.062, *η*^2^ = 0.019, and Supplement or Alternative to Medical Care (SAMC) showed a marginal trend, *F* (2, 297) = 2.46, *p* = 0.087, *η*^2^ = 0.016. No significant effect was found for information seeking (IS), *F* (2, 297) = 1.81, *p* = 0.165, *η*^2^ = 0.012. Tukey HSD indicated that the AI channel produced significantly higher source credibility than the short-video condition (*p* = 0.004) and marginally higher than the text-based condition (*p* = 0.057). For perceived usefulness, both AI vs. text-based (*p* = 0.039) and AI vs. short-video (*p* = 0.036) contrasts were significant, again favoring the AI channel.

#### 3.2.3. Structural Equation Modeling

**Figure 5:**
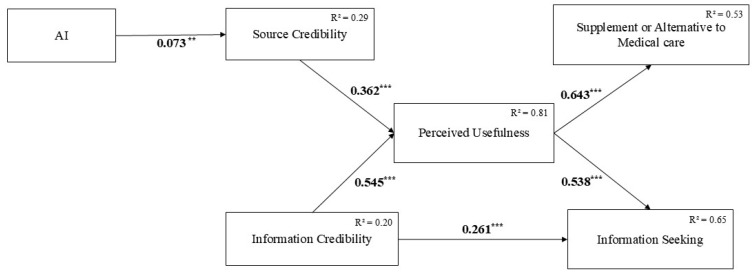
Structural Equation Model of Channel, Credibility, Usefulness and OHISB (Study 2). Note. All paths are standardized. Black lines indicate significant paths (*p* < 0.05). Only statistically significant paths (*p* < 0.05) are displayed as directional arrows. Nonsignificant paths were estimated in the model but omitted from the figure to maintain visual clarity. Covariances among variables were estimated but not depicted in the figure to maintain clarity. Age, gender, eHealth literacy, and health anxiety were included as covariates in the model. ** *p* < 0.01. *** *p* < 0.001.

A structural equation model was constructed using Study 2 data to test how exposure to different channels influenced behavioral outcomes through credibility and perceived usefulness, using the text-based condition as the reference category. This model followed the same theoretical structure as in Study 1 but with one necessary adaptation: the Support Seeking subscale was omitted because AI-based channels do not involve social support functions (e.g., peer interaction, emotional support), making the construct conceptually inapplicable in this experimental context. All hypothesized direct paths were estimated in the model. The model (Figure 5) fit data well, *χ*^2^ = 13.35, *df* = 7, *p* = 0.064, CFI = 0.996, TLI = 0.980, RMSEA = 0.056 [0.000, 0.100], and SRMR = 0.033. In Figure 5, only statistically significant paths (*p* < 0.05) are displayed as directional arrows; nonsignificant paths were estimated but omitted from the figure to maintain visual clarity. The effect of AI channel on source credibility was significant (*β* = 0.073, *p* = 0.006), whereas direct effects of channel type on information credibility, SAMC, and IS were nonsignificant (all *ps* > 0.05).

Table 6 shows the standardized path coefficients in the final model. The effect of AI channel on source credibility was significant (*β* = 0.073, *p* = 0.006), indicating that exposure to AI-based channels led to higher perceived source credibility compared with the text-based condition. Both source credibility (*β* = 0.362 ***, *p* < 0.001) and information credibility (*β* = 0.545 ***, *p* < 0.001) positively predicted perceived usefulness, and perceived usefulness in turn strongly predicted SAMC (*β* = 0.643 ***, *p* < 0.01) and IS (*β* = 0.538 ***, *p* < 0.01). These findings indicate that AI exposure increased perceived source credibility, which subsequently enhanced perceived usefulness and, indirectly, health-information-seeking behaviors. Bootstrap analyses of indirect effects (Table 6) showed that the indirect effect from AI to IS, and from AI to SAMC were significant, indicating that AI exposure enhances source credibility, which subsequently promotes perceived usefulness and strengthens both information-seeking and alternative medical-care-seeking intentions.

## 4. Discussion

This research extends the CMIS framework in three specific ways and provides a coherent picture of how modern online channels influence credibility perceptions and online health information-seeking behaviors (OHISB). First, while prior extensions have focused on individual antecedents (e.g., demographic factors, health literacy), we systematically incorporate channel type (AI-based, short-video, text-based) as a formal antecedent within the CMIS structure. Second, integrating CMIS with the IAM clarifies a sequential mediation pathway—channel characteristics shape credibility perceptions, which inform perceived usefulness, which in turn drives behavioral intentions—rather than treating these as parallel predictors. Third, our multi-method approach (pilot validation, cross-sectional survey, and experiment) provides stronger construct validation than prior single-study extensions.

The pilot validation (Appendix A) established the Chinese version of the OHISB scale and confirmed its three-factor structure. Study 1 then tested the extended CMIS framework by examining the relationships among channel type, source and information credibility, perceived usefulness, and behavioral outcomes in a correlational design. Study 2 provided a more stringent test by experimentally manipulating channel type while holding information content constant, offering stronger evidence about how causal exposure can shape credibility perceptions and downstream intentions.

The divergent findings between Study 1 and Study 2 should be interpreted in light of two interrelated factors rather than as a theoretically driven comparison. First, methodological improvement distinguishes the studies. Study 1 relied on participants’ recall and reconstruction of channel experiences, which introduces memory-based biases and activates generalized stereotypes. Study 2 addressed this limitation through controlled experimental exposure to standardized stimuli, capturing immediate reactions without retrospective reconstruction. Methodological research has documented that recall-based assessments and direct exposure paradigms can produce divergent evaluations (Schwarz, 2007). However, we caution against over-interpreting this contrast as a theoretically meaningful “recall versus direct exposure” experimental paradigm; rather, this represents methodological refinement to address Study 1’s limitations. Second, temporal–cultural context differed between studies. Study 1 was conducted prior to widespread public familiarity with conversational AI technologies in China, whereas Study 2 followed their rapid cultural diffusion (including the widespread adoption of DeepSeek). This temporal distinction suggests that participants in Study 2 approached AI-presented information with greater baseline familiarity, potentially amplifying positive immediate reactions. The observed pattern—neutral AI evaluations in Study 1 but favorable AI credibility in Study 2—likely stems from an interaction between these factors. Study 2’s methodological rigor (direct exposure) captured favorable immediate reactions that were inaccessible to Study 1’s recall-based design, while the temporal shift to a period of greater AI familiarity may have lowered initial skepticism. We emphasize that Study 1 provides preliminary baseline attitudes (with acknowledged limitations), while Study 2 provides more reliable causal evidence obtained at a different cultural moment. Importantly, these explanations are post hoc and speculative, and were not directly measured in the current studies; future research could adjudicate between them by directly measuring AI familiarity and trust.

Perceived usefulness consistently emerged as a pivotal cognitive mediator linking credibility to behavior. This pattern aligns with the Information Adoption Model (IAM) (Cheung et al., 2008; Sussman & Siegal, 2003), emphasizing that individuals’ judgments of usefulness transform credibility beliefs into action tendencies. Moreover, the dissociation between source credibility (trust in the communicator) and information credibility (trust in message accuracy) highlights two complementary pathways: relational trust motivating supportive and alternative behaviors, and informational trust driving analytic, information-seeking actions. Wathen and Burkell (2002) emphasized that users often evaluate credibility through dual-process mechanisms—rapid, affective judgments about the source and more deliberative evaluations of message content—while Hilligoss and Rieh (2008) further integrated these perspectives into a unified framework of credibility assessment that accounts for contextual, cognitive, and social cues. These frameworks provide a theoretical foundation for interpreting the differentiated roles of source and information credibility observed in the present research. Together, these findings refine the Comprehensive Model of Information Seeking (CMIS) by revealing that channel features operate through layered credibility evaluations before influencing behavioral engagement.

While the present study establishes baseline differences in channel credibility and usefulness perceptions, we acknowledge that these effects may be moderated by individual difference variables not systematically examined here. Recent research indicates that demographic characteristics such as age and gender can significantly moderate user satisfaction and usage intentions toward AI-driven health chatbots, with distinct patterns emerging across different population segments (Alzahrani & Weerakkody, 2026). Furthermore, individual capabilities such as e-health literacy have been shown to moderate the relationship between online health information-seeking behaviors and health outcomes (Peimani et al., 2024), suggesting that the effectiveness of channel-based interventions may vary by users’ competencies in evaluating digital health content. Additional factors such as prior experience with emerging technologies, digital self-efficacy, and the severity of health concerns may also shape how individuals respond to different information channels. These potential moderating factors represent important boundary conditions that future research should systematically investigate through moderated experimental designs or multi-group structural analyses.

### 4.1. Theoretical and Practical Implications

The present research extends existing theory in several ways. It demonstrates that the type of channel itself—text, short-video, or AI—functions as an antecedent within the CMIS framework, shaping both cognitive and behavioral processes. Integrating the CMIS with the IAM clarifies a sequential pathway in which credibility informs perceived usefulness, which in turn predicts OHISB. Distinguishing between source and information credibility further enhances the conceptual precision of credibility research and explains why different health behaviors may arise from distinct cognitive routes. These theoretical refinements collectively broaden the scope of traditional health information models, adapting them to the realities of multimodal and AI-mediated communication.

From an applied perspective, the results suggest that AI-based health communication holds strong potential for promoting user engagement through higher perceived credibility and usefulness. However, this benefit must be balanced with caution against over-trust in AI outputs. Increased perceived credibility carries risks of misinformation amplification—users may accept and share AI-generated content without independent verification—and over-reliance, where individuals delay seeking professional medical advice based on AI recommendations alone. To mitigate these risks, designers should integrate transparency indicators (e.g., confidence scores, source attribution) and verification prompts that encourage users to cross-check critical health information with qualified professionals. In parallel, improving eHealth literacy remains crucial, as individuals with higher literacy displayed more adaptive credibility assessments and more deliberate information-seeking behaviors across studies.

### 4.2. Limitations and Future Directions

Several limitations warrant attention. First, all behavioral measures relied on self-report, which may introduce common method bias and limit ecological validity. The exclusive use of self-report for both predictors (credibility perceptions) and outcomes (behavioral intentions) may inflate observed relationships due to shared measurement method rather than true associations. Future research should employ computer-interactive behavioral assessments (e.g., clickstream tracking, eye-tracking) to capture actual engagement and reduce method bias. Second, the present work was conducted in a single cultural context; replication across cultural settings will clarify generalizability. Third, although eHealth literacy and health anxiety were treated as covariates, their potential moderating effects merit examination—particularly whether high-anxiety users might over-rely on AI advice or high-literacy individuals might adopt more evaluative strategies. Fourth, the framework may appear oversimplified for a conditional, context-dependent phenomenon. Individual factors (age, digital literacy, prior AI experience, health issue severity) likely moderate channel effects, but these relationships were not fully explored. Additionally, findings may vary across methodological and contextual factors: the experimental single-exposure paradigm may not reflect naturalistic repeated use; AI credibility advantages may reflect temporary novelty effects; the specific chatbot interface may not generalize to other AI formats; and the mild health topics employed may favor AI channels more than severe or emotionally charged conditions. These boundary conditions qualify generalizability and highlight priorities for future research, including moderated experimental designs and multi-group analyses. Fifth, the sampling strategy focused on highly educated Chinese adults, which limits generalizability to populations with lower educational attainment or reduced digital access. This demographic profile is theoretically relevant: AI-driven health information tools, particularly during their early diffusion stages, present non-trivial learning thresholds that affect adoption patterns. At the time of data collection, conversational AI platforms were in a relatively early phase of public adoption, and highly educated individuals likely represented “early adopters” equipped to navigate these novel interfaces. Consequently, the observed AI credibility advantages may represent a “best-case” scenario among tech-savvy users. As AI interfaces become more ubiquitous and user-friendly, effects may shift to benefit different segments—particularly individuals with lower health literacy who may value AI’s ability to simplify complex information, or conversely, populations that remain underserved due to persistent digital divides. Future research must deliberately extend to these populations as AI health tools transition from early adoption to mainstream use (Liu et al., 2024; Tennant et al., 2015). Sixth, the observed AI credibility advantages should be interpreted within the scope of the experimental conditions. The study employed brief, single-exposure presentations of mild-to-moderate health topics under controlled laboratory conditions. These findings do not constitute endorsement of AI as a universally reliable health information source, nor do they generalize to high-stakes medical decisions, emergency contexts, or complex clinical scenarios requiring professional judgment. Users should maintain critical evaluation skills alongside AI-assisted information seeking. Seventh, AI credibility ratings may reflect both surface-level features (interface appeal, structured formatting, conversational modernity) and deeper assessments of medical reliability; these dimensions were not disentangled in the present study. Future research should employ multi-dimensional measures to clarify whether observed advantages stem from experiential appeal or substantive trust in medical accuracy. Additionally, while channel type was experimentally manipulated, the mediators (credibility perceptions, perceived usefulness) were measured rather than manipulated. This limits strong causal claims about the full mediation chain. Finally, longitudinal replication of both studies would help disentangle methodological effects from evolving cultural familiarity with AI technologies, determining whether the observed channel credibility rankings remain stable as AI tools mature and public familiarity stabilizes.

This research advances theoretical and empirical understanding of OHISB by demonstrating that perceived credibility and usefulness act as psychological bridges linking information-channel characteristics to digital health engagement. The results demonstrate that manipulated AI channel conditions enhance source credibility and perceived usefulness, thereby strengthening indirect pathways to both information-seeking and alternative-medical intentions, whereas information credibility exerts an additional direct influence on information seeking. These findings enrich the credibility–usefulness pathway central to the extended CMIS, highlight the nuanced functional roles of different credibility dimensions, and provide actionable implications for designing trustworthy, effective, and ethically responsible AI-based health-communication systems.

## Figures and Tables

**Table 1:** Descriptive Statistics, Cronbach’s α and Correlations (Study 1).

Measures	*M*	*SD*	1	2	3	4	5	6	7	8	9
1. EHL	5.72	0.66	**0.66**								
2. SHAI	2.29	0.56	0.01	**0.91**							
3. SC	5.38	0.94	0.66 ***	−0.06	**0.88**						
4. IC	5.55	0.87	0.67 ***	−0.07	0.86 ***	**0.78**					
5. PU	5.68	0.77	0.65 ***	−0.03	0.75 ***	0.78 ***	**0.82**				
6. OHISB	5.12	0.72	0.56 ***	0.17 **	0.56 ***	0.51 ***	0.53 ***	**0.88**			
7. SS	5.36	0.99	0.60 ***	0.08	0.55 ***	0.51 ***	0.48 ***	0.81 ***	**0.90**		
8. SAMC	4.10	1.26	0.20 ***	0.17 **	0.30 ***	0.23 ***	0.27 ***	0.78 ***	0.34 ***	**0.86**	
9. IS	5.81	0.61	0.43 ***	0.15 **	0.39 ***	0.43 ***	0.52 ***	0.57 ***	0.34 ***	0.25 ***	**0.77**

Note. ** *p* < 0.01. *** *p* < 0.001. Cronbach’s α for each scale is in bold. EHL = Electronic Health Literacy; SHAI = Health Anxiety Inventory; SC = Source Credibility; IC = Information Credibility; PU = Perceived Usefulness; OHISB = Online Health Information-Seeking Behavior; SS = Support Seeking; SAMC = Supplement or Alternative to Medical Care; IS = Information Seeking. Discriminant validity between SC and IC was further examined using AVE; the conventional criterion was not fully met, AVE_SC = 0.652 and AVE_IC = 0.546. Although the AVE criterion was not fully met (Fornell & Larcker, 1981), SC and IC showed differential predictive patterns in subsequent structural equation modeling analyses.

**Table 2:** One-Way ANOVA Testing Channel Differences (Study 1).

Measures	Text	Short-Video	AI	*F* (*df*_1_, *df*_2_)	*p*	*η* ^2^
	*M* (*SD*)	*M* (*SD*)	*M* (*SD*)			
SC	5.43 (0.83)	5.30 (1.03)	5.39 (0.94)	0.492 (2, 297)	0.612	0.003
IC	5.60 (0.79)	5.46 (0.95)	5.60 (0.86)	0.861 (2, 297)	0.424	0.006
PU	5.69 (0.64)	5.66 (0.84)	5.68 (0.83)	0.033 (2, 297)	0.968	0.000
SS	5.50 (0.79)	5.35 (1.07)	5.24 (1.06)	1.791 (2, 297)	0.169	0.012
SAMC	4.40 (1.10)	4.12 (1.30)	3.78 (1.31)	6.180 (2, 297)	0.002 **	0.040
IS	5.83 (0.61)	5.83 (0.62)	5.78 (0.62)	0.208 (2, 297)	0.813	0.001

Note. ** *p* < 0.01. SC = Source Credibility. IC = Information Credibility. PU = Perceived Usefulness. SS = Support Seeking; SAMC = Supplement or Alternative to Medical Care. IS = Information Seeking.

**Table 3:** Standardized Path Coefficients for the Structural Equation Model (Study 1).

Paths	*β*	SE	*z*	*p*	95% CI
Channel → Credibility					
Short-Video → SC	−0.008	0.099	−0.158	0.874	[−0.208, 0.180]
AI → SC	−0.016	0.102	–0.309	0.757	[−0.224, 0.178]
Short-Video → IC	0.002	0.092	0.036	0.972	[−0.174, 0.184]
AI → IC	0.018	0.092	0.359	0.719	[−0.145, 0.220]
Credibility → Usefulness					
SC → PU	0.248	0.082	2.484	0.013	[0.043, 0.370]
IC → PU	0.439	0.077	5.088	<0.001	[0.244, 0.543]
Usefulness → Behavioral					
PU → SS	−0.004	0.127	−0.043	0.966	[−0.252, 0.241]
PU → SAMC	0.178	0.135	2.146	0.032	[0.016, 0.550]
PU → IS	0.408	0.095	3.415	<0.001	[0.148, 0.520]
Credibility → Behavioral					
SC → SS	0.293	0.108	2.873	0.004	[0.111, 0.536]
SC → SAMC	0.326	0.137	3.216	0.001	[0.181, 0.716]
SC → IS	−0.117	0.070	−1.102	0.271	[−0.206, 0.066]
IC → SS	−0.010	0.154	−0.070	0.944	[−0.305, 0.297]
IC → SAMC	−0.150	0.161	−1.350	0.177	[−0.549, 0.091]
IC → IS	0.114	0.080	1.009	0.313	[−0.080, 0.237]

Note. Text-based was the reference channel; Short-Video = Short-Video vs. Text-based, AI = AI vs. Text-based. Age, gender, eHealth literacy and health anxiety were controlled but not shown for parsimony. Only paths relevant to the theoretical model are presented. Standardized coefficients (*β*) are reported.

**Table 4:** Descriptive Statistics, Cronbach’s α and Correlations (Study 2).

Measure	*M*	*SD*	1	2	3	4	5	6	7	8
1. EHL	5.69	0.68	**0.70**							
2. SHAI	2.43	0.54	−0.13 *	**0.91**						
3. SC	5.27	1.00	0.49 ***	0.38 ***	**0.91**					
4. IC	5.44	1.01	0.41 ***	0.38 ***	0.87 ***	**0.84**				
5. PU	5.58	0.96	0.44 ***	0.47 ***	0.84 ***	0.88 ***	**0.88**			
6. OHISB	5.21	1.02	0.46 ***	0.49 ***	0.76 ***	0.78 ***	0.82 ***	**0.93**		
7. SAMC	4.80	1.26	0.43 ***	0.38 ***	0.67 ***	0.68 ***	0.72 ***	0.94 ***	**0.90**	
8. IS	5.62	0.95	0.42 ***	0.56 ***	0.74 ***	0.77 ***	0.80 ***	0.89 ***	0.69 ***	**0.90**

Note. * *p* < 0.05. *** *p* < 0.001. Cronbach’s α for each scale is in bold. EHL = Electronic Health Literacy; SHAI = Health Anxiety; SC = Source Credibility; IC = Information Credibility; PU = Perceived Usefulness; OHISB = Overall Online Health Information-Seeking Behavior; SAMC = Supplement or Alternative to Medical Care; IS = Information Seeking. Discriminant validity between SC and IC was further examined using AVE; the conventional criterion was not fully met, AVE_SC = 0.704 and AVE_IC = 0.635. Although the AVE criterion was not fully met (Fornell & Larcker, 1981), SC and IC showed differential predictive patterns in subsequent structural equation modeling analyses.

**Table 5:** One-Way ANOVA Testing Channel Differences (Study 2).

Measures	Text	Short-Video	AI	*F* (*df*_1_, *df*_2_)	*p*	*η* ^2^
	*M* (*SD*)	*M* (*SD*)	*M* (*SD*)			
SC	5.21 (0.93)	5.08 (1.17)	5.53 (0.81)	5.56 (2, 297)	0.004 **	0.036
IC	5.37 (1.01)	5.31 (1.15)	5.63 (0.81)	2.81 (2, 297)	0.062 ^+^	0.019
PU	5.47 (1.05)	5.47 (1.04)	5.80 (0.73)	4.07 (2, 297)	0.018 *	0.027
SAMC	4.74 (1.31)	4.64 (1.34)	5.03 (1.11)	2.46 (2, 297)	0.087 ^+^	0.016
IS	5.56 (1.06)	5.52 (0.99)	5.76 (0.77)	1.81 (2, 297)	0.165	0.012

Note. + *p* < 0.10. * *p* < 0.05. ** *p* < 0.01. SC = Source Credibility. IC = Information Credibility. PU = Perceived Usefulness. SAMC = Supplement or Alternative to Medical Care. IS = Information Seeking.

**Table 6:** Standardized Path Coefficients, Indirect and Total Effect of AI on OHISB (Study 2).

Path	*β*	SE	*z*	*p*	95% CI
AI → SC	0.073	0.027	2.728	0.006	[0.021, 0.125]
SC → PU	0.362	0.057	6.357	<0.001	[0.249, 0.465]
IC → PU	0.545	0.066	8.258	<0.001	[0.422, 0.667]
PU → SAMC	0.643	0.073	8.836	<0.001	[0.496, 0.783]
PU → IS	0.538	0.062	8.667	<0.001	[0.413, 0.656]
IC → IS	0.261	0.080	3.262	0.001	[0.093, 0.398]
Ind (AI → SC → PU → IS)	0.029	0.013	2.166	0.030	[0.008, 0.062]
Ind (AI → SC → PU → SAMC)	0.045	0.020	2.318	0.020	[0.013, 0.090]
Total (AI → IS)	0.029	0.013	2.166	0.030	[0.008, 0.062]
Total (AI → SAMC)	0.045	0.020	2.318	0.020	[0.013, 0.090]

Note. Age, gender, eHealth literacy, and health anxiety were controlled as covariates. Text-based channel served as the reference condition. AI = AI vs. Text-based (reference). SC = source credibility; IC = information credibility; PU = perceived usefulness; SAMC = supplement or alternative to medical care; IS = information seeking; Ind = indirect effect; Total = total effect. Bootstrap 95% confidence intervals (CIs) are reported.

## Data Availability

The data presented in this study are available on request from the corresponding author due to privacy and ethical restrictions.

## Supplementary Materials

The following supporting information can be downloaded at: https://www.mdpi.com/article/10.3390/bs16071137/s1.

## Appendix A. OHISB Scale (Chinese Version) and Validation Summary

### Appendix A.1. Scale and Response Format

Online health information-seeking behavior (OHISB) was measured using an adapted Chinese version of the OHISB scale developed by Popovac and Roomaney (2022). Participants responded on a 7-point Likert scale ranging from 1 (strongly disagree) to 7 (strongly agree). Following the validation procedure described below, a refined 20-item version was retained, comprising three dimensions: Support Seeking (SS 8 items), Supplement or Alternative to Medical Care (SAMC 6 items) and information seeking (IS 6 items). Higher scores indicate greater engagement in the corresponding type of online health information-seeking behavior.

### Appendix A.2. Translation and Adaptation

The OHISB items were translated into Chinese and then back-translated by bilingual researchers, with discrepancies resolved through discussion. Participants completed the Chinese version. English wording is presented for reporting purposes.

### Appendix A.3. Pilot Validation Summary

A pilot validation sample included 258 working adults or retirees (168 women, 65.1%; age 20–65 years, *M* = 30.87, *SD* = 8.62) recruited via the Credamo platform. An exploratory factor analysis (principal-axis factoring with oblimin rotation) on the original 37 items supported a three-factor solution (*KMO* = 0.944; Bartlett’s test *p* < 0.001). After removing items with low primary loadings (<0.40), substantial cross-loadings (>|0.30|), or conceptual redundancy, 20 items were retained. The three factors (SS, SAMC, IS) explained 54.22% of the total variance, and retained item loadings were all ≥0.50.

A confirmatory factor analysis supported the three-factor model with good fit: *χ*^2^(167) = 288.93, *p* < 0.001, *CFI* = 0.957, *TLI* = 0.951, *RMSEA* = 0.053 (90% CI [0.043, 0.063]), and *SRMR* = 0.062. Internal consistency for the overall 20-item scale was excellent (Cronbach’s *α* = 0.909).

### Appendix A.4. Final Item List

The final 20 items are presented in Table A1.

**Table A1:** Factor Loadings and Cronbach’s α for the Three-Factor Model of the OHISB Scale.

Subscales	#	Item Description	$\lambda_{EFA}$	$\lambda_{CFA}$
SS *α* = 0.93	1	I turn to other online users in order to connect with others with similar health concerns.	0.80	0.81
	2	I use OSGs to feel less isolated.	0.70	0.75
	3	I engage in health support groups and forums in order to learn about the experiences of others.	0.90	0.87
	4	I engage in health-related discussion forums for support.	0.80	0.84
	5	I turn to other online users in order to give emotional support to others with similar health concerns.	0.81	0.81
	6	I use OSGs so that I don’t feel as if I am the only one with my illness.	0.66	0.70
	7	I feel comfortable expressing my views in OSGs.	0.80	0.79
	8	I turn to other online users in order to give information and advice to others with similar health concerns.	0.73	0.79
SAMC *α* = 0.88	9	I turn to other online users for information about alternatives to medication prescribed by a doctor.	0.72	0.80
	10	I considered changing my medication as a result of experiences others have told me about online.	0.88	0.90
	11	I have changed or stopped my medication due to information I found on the internet.	0.85	0.84
	12	I considered changing my treatment as a result of the experiences others have told me about online.	0.82	0.79
	13	I consult the internet when I want to decide whether to follow a doctor’s recommended treatment.	0.52	0.63
	14	I searched for health-related information online because I did not have time to visit a doctor.	0.54	0.47
IS *α* = 0.80	15	I seek information on the internet related to an illness that I am experiencing.	0.56	0.58
	16	I use the internet to search for a diagnosis for symptoms I may be experiencing.	0.71	0.69
	17	I use the internet to search for general information related to my health.	0.55	0.56
	18	I consult the internet when I experience symptoms that worry me.	0.71	0.69
	19	I use the internet to diagnose myself.	0.61	0.69
	20	I use the internet to seek health-related information prior to consulting a medical professional.	0.59	0.61

Note. All factor loadings are standardized. SS = Support Seeking; SAMC = Supplement or Alternative to Medical Care; IS = Information Seeking.
