# Supplementary material for: Channel Effects on Online Health Information Seeking in the Age of AI: An Extension of the CMIS Framework
Source: Behav Sci (Basel). 2026 Jul 7;16(7):1137. doi: 10.3390/bs16071137 (PMC13403515; doi:10.3390/bs16071137)
Supplement: Supplementary file 1 [file behavsci-16-01137-s001.zip › behavsci-4227984-supplementary.pdf]

Supplemental Material: Experimental Stimuli

This document provides detailed descriptions of the experimental stimuli used in Study 2. All three conditions presented identical health information content in Chinese, adapted from authoritative medical sources. The two topics were: (1) *Can ibuprofen and aspirin be taken together?* and (2) "*How should acute gastroenteritis be treated?* Only the presentation format varied by channel type.

1. AI Channel Condition

Participants viewed an AI chatbot interface displaying a conversational interaction between a user and an AI health assistant. The stimulus presented: A user query asking about the health topic (e.g., "*Can I take ibuprofen and aspirin together?*"); AI-generated responses providing structured health information in a dialogue format; Visual design, featuring clean dialogue bubbles, professional formatting, and a simulated web-interface layout.

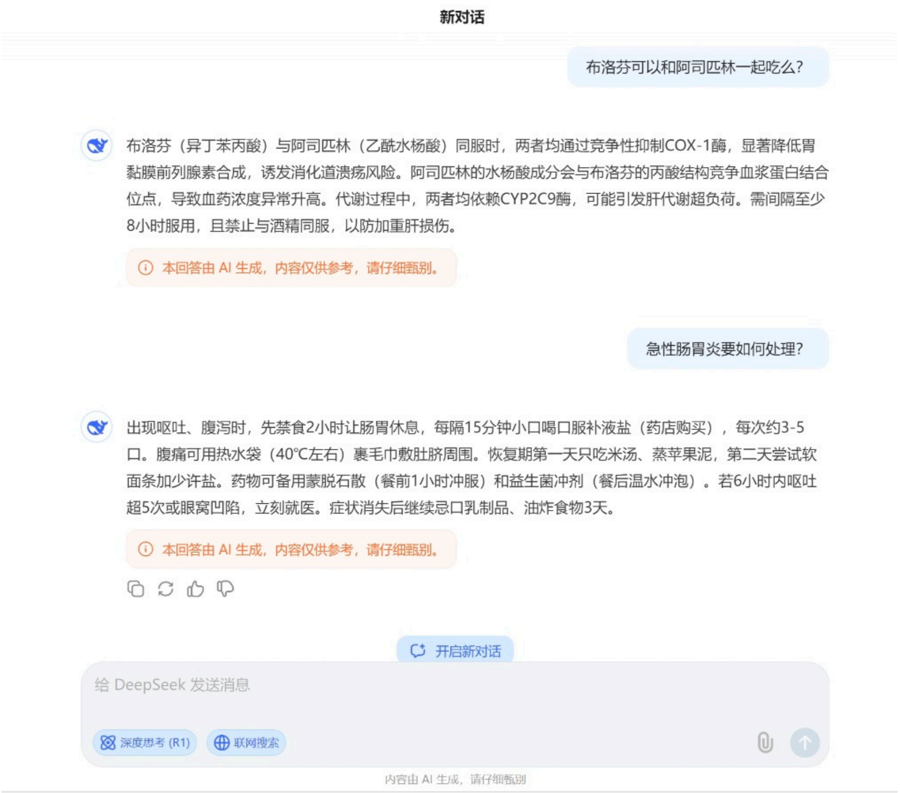

2. Short-Video Channel Condition

Participants viewed content presented in a short-video platform format, specifically designed to resemble health content on Chinese mini-program video platforms. The stimulus featured:

A video player interface with health information presented as video content; Visual layout optimized for mobile vertical viewing (9:16 aspect ratio); Platform-style elements including video thumbnails, brief descriptions, and interface cues typical of short-video health content.

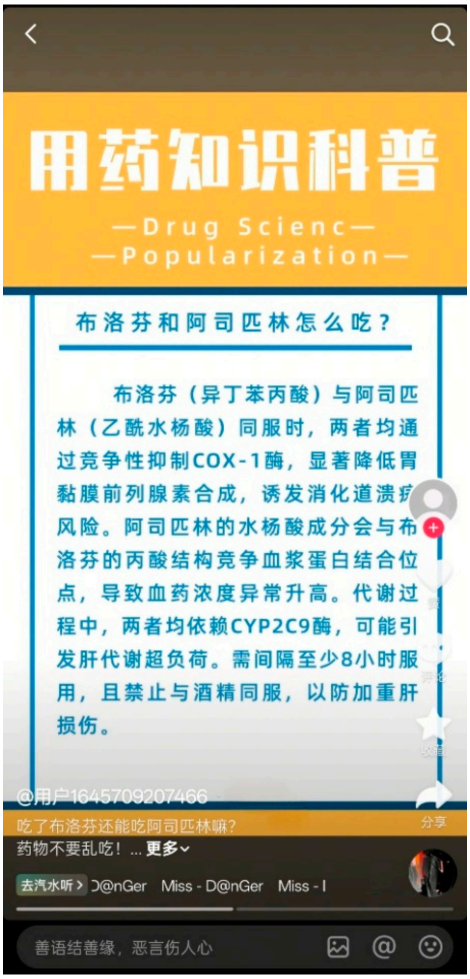

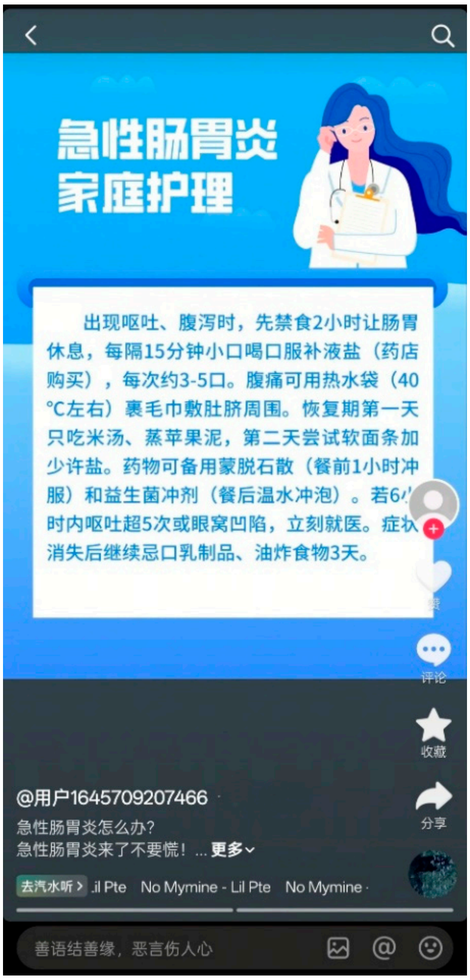

3. Text-Based Channel Condition

Participants viewed content presented as social media posts on a popular Chinese lifestyle-sharing platform. The stimulus consisted of: Text accompanied by relevant images in a social media post layout; Informal, community-style presentation typical of health discussions on social platforms, including user avatars, engagement metrics, and comment sections.

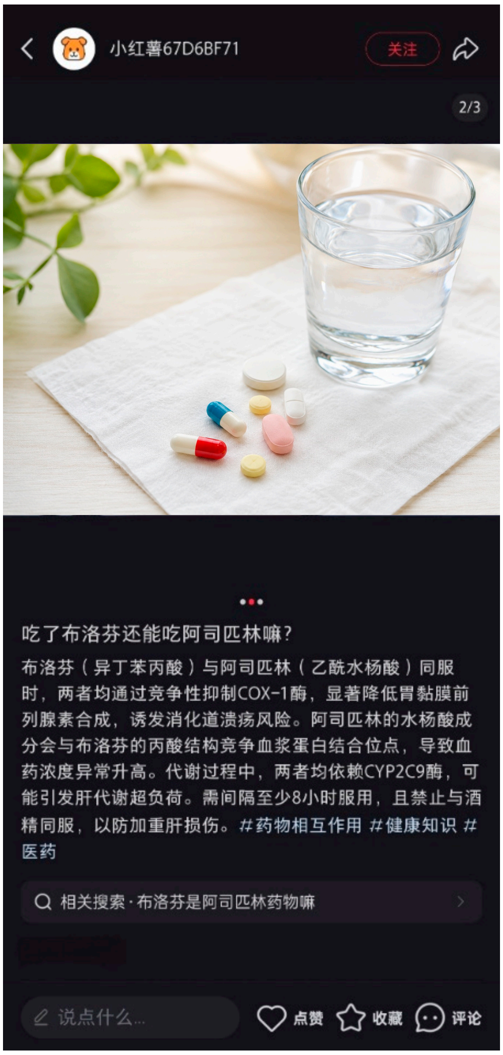

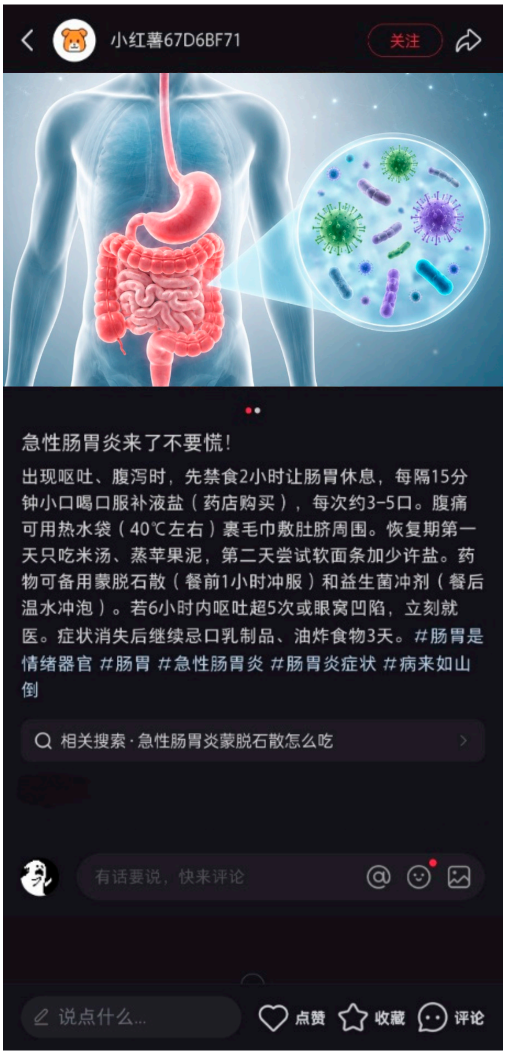

Across all three conditions, the core health information content (facts, recommendations, medical accuracy) remained identical to ensure that observed differences in credibility perceptions could be attributed to channel presentation format rather than information content. The stimuli were designed to closely mirror the actual interfaces that Chinese participants encounter in their daily online health information seeking, while maintaining experimental control over content equivalence.

The experimental stimuli were originally created and presented in Chinese to Chinese-speaking participants. The English translations provided below are for editorial and review purposes only; the actual materials seen by participants were in Chinese. Across all three channel conditions (AI, short-video, and text-based), the core health information content was identical in the original Chinese wording, with only the presentation format and interface

design varying by condition. The two health topics and their complete content are translated as follows:

Topic 1: Can ibuprofen and aspirin be taken together?

Ibuprofen (isobutylphenyl propionic acid) and aspirin (acetylsalicylic acid) both act by competitively inhibiting the COX-1 enzyme. When taken simultaneously, they significantly reduce gastric mucosal prostaglandin synthesis, increasing the risk of gastrointestinal ulcers. The salicylic acid component of aspirin competes with the propionic acid structure of ibuprofen for plasma protein binding sites, leading to abnormally elevated blood drug concentrations. Both drugs rely on the CYP2C9 enzyme during metabolism, which may trigger hepatic metabolic overload. They should be taken at least 8 hours apart, and alcohol must be avoided to prevent exacerbating liver damage.

Topic 2: How should acute gastroenteritis be treated?

When vomiting and diarrhea occur, fast for 2 hours first to let the gastrointestinal tract rest. Then drink oral rehydration salts (purchased at pharmacies) in small sips every 15 minutes, approximately 3–5 sips each time. For abdominal pain, apply a hot water bag (around 40 ℃) wrapped in a towel to the area around the navel. On the first day of recovery, consume only rice soup and steamed apple puree; on the second day, try soft noodles with a small amount of salt. Medications that can be kept on hand include montmorillonite powder (take 1 hour before meals) and probiotic powder (dissolve in warm water after meals). If vomiting occurs more than 5 times within 6 hours or if the eye sockets appear sunken, seek medical attention immediately. After symptoms disappear, continue avoiding dairy products and fried foods for 3 days.
